# Developing and evaluating a peer-based mental health literacy intervention with adolescent athletes

**DOI:** 10.1371/journal.pone.0274761

**Published:** 2022-12-15

**Authors:** Michael Panza, Grace Redman, Matthew Vierimaa, Stewart A. Vella, Melissa Bopp, M. Blair Evans

**Affiliations:** 1 Department of Kinesiology, Pennsylvania State University, University Park, PA, United States of America; 2 School of Kinesiology, Acadia University, Wolfville, NS, CAN; 3 School of Psychology, University of Wollongong, Wollongong, NSW, AUS; 4 Department of Psychology, Western University, London, ON, CAN; Belgrade University Faculty of Medicine, SERBIA

## Abstract

Widespread adolescent involvement in organized sport means that sport contexts are well-suited to ‘actively’ integrate prevention programs that may promote population-level change. This mixed methods study aimed to evaluate the feasibility and acceptability of a peer-based mental health literacy intervention. The intervention (i.e., Team Talk) was presented to eleven adolescent sport teams in the United States, with a total of 174 participants. Athlete participants completed surveys immediately before and after the intervention—including measures of workshop acceptability, social identity, and help-seeking behaviors. Semi-structured interviews were also conducted with a subset of five athletes, nine parents, and two coaches. With respect to recruitment as an indicator of feasibility, club-level adoption of the intervention was low, with difficulty recruiting clubs for intervention delivery. This signals that feasibility of the intervention–as it is currently designed and implemented by the research team–is low when considering similar competitive adolescent sport clubs and delivered as team-level workshops. Meanwhile, participants reported high acceptability of the intervention, and acceptability levels across participants was predicted by contextual factors related to implementation such as session duration. Regarding limited efficacy testing with measures completed before and after the intervention session: (a) social identity scores increased following the intervention, and (b) significant differences were not identified regarding efficacy to recognize symptoms of mental disorders. Athlete, coach, and parent interview responses also described potential adaptations to mental health programs. This research demonstrates the potential utility of peer-based mental health literacy interventions, while also revealing that further implementation research is necessary to adapt mental health literacy interventions to suit diverse adolescent sport contexts.

## Introduction

Among numerous positive and negative experiences throughout adolescence, mental health problems are–for many individuals from 12 to 18 years of age–one common aspect of the transition into adulthood. Most people who are diagnosed with mental health disorders will receive their first diagnoses before the age of 18, with anxiety and depression being key concerns during early and middle adolescence [1, 2]. For example, around 30% of Americans between the ages of 12 and 18 years will experience symptoms of depression in a given year [3]. Mental health problems related to anxiety and depression symptoms are also critical to consider because of their cascading effects through significant economic, personal, and social costs that can last the entirety of one’s life [4]. These significant costs highlight the critical need for mental health-related interventions during adolescence.

Mental health literacy programs are increasingly evident in sport settings [5], with numerous characteristics of sport signaling that it can be a promising context. One reason why organized sport is a promising context is because young athletes often tacitly gain mental health benefits through their involvement. Considering the results of longitudinal studies regarding sport participation, youth who participate in sport report fewer mental health problems along with increased markers of positive youth development, when compared to youth who do not participate in sport [6]. Athletes may also face conditions in teams and clubs that either harm their mental health, or that construct a stigma around mental health that effectively omits the topic from sport settings. Each of these justifications provide support for implementing preventative educational interventions around mental health within sport. Recent evidence indeed signals that sport-based interventions can meet goals of preventing mental disorders and promoting mental health (e.g., [7]).

Sport is also a promising setting for preventive interventions because it is so prevalent; many adolescents regularly engage in sport or physical activity programs [8]. The prevalence of sport allows for mental health messaging that can be delivered in ways that complement messages from other community contexts. This aligns with the value of multi-level prevention approaches that are valued in broader frameworks regarding community-based health promotion [9], as well as frameworks regarding promoting mental health in communities (i.e., mental health literacy [10]). Indeed, various settings (e.g., schools, social media) are increasing adolescents’ exposure to mental health literacy content. However, it is critical to reinforce such messages through specific contexts like sport to inform adolescents of the relative commonality of mental health problems and suggest peers as supportive resources. Therefore, we conducted the current research to develop and pilot an intervention that targeted components of athletes’ peer environments expected to amplify intervention goals.

### Promoting mental health literacy through sport

Mental health literacy is the guiding framework for several recent sport-based interventions to promote mental health (e.g., [7, 11, 12]). Mental health literacy refers to individuals’ knowledge relating to mental disorders, and it is expected that ‘literate’ adolescents will better recognize and manage problems. Jorm and colleagues’ [10] framework specifically highlights that mental health literacy spans: disorder recognition, help-seeking and self-help knowledge, prevention strategies, and awareness of professional treatment. A key goal for targeting mental health literacy is to empower the public with knowledge regarding the wellbeing of themselves and others [13]. An approach grounded in mental health literacy acknowledges that people are commonly unaware of available mental health resources, avoid seeking treatment, and may not be aware of how to recognize problems [13].

Interventions have effectively improved mental health literacy in varying youth settings, including high school students [14] as well as broader samples of adolescents and young adults sampled from the community [15]. Recent sport-based interventions with adolescents also apply Jorm’s [13] mental health literacy framework. Vella et al. [11] sought to determine the effectiveness of this strategy through a multi-component program named *Ahead of the Game*. This program aimed to increase the mental health literacy of male adolescent athletes and their support systems (i.e., parents and coaches).

The *Ahead of the Game* program also intervened-upon group environments through a workshop termed *Help Out a Mate*. This 45-minute workshop was delivered to individual teams with all consenting team members present in a single session (i.e., single-team sessions) and focused on increasing knowledge of mental health problems and intentions to provide and seek help for those experiencing such concerns [11]. Liddle et al. [7] conducted a randomized-control trial with a sample of 102 adolescent male athletes across nine teams, assessing the effects of the *Help Out a Mate* intervention compared to a waitlist control. Although athletes’ attitudes did not shift regarding some outcomes (e.g., help-seeking intentions), increases were evident for several outcomes at a one-month follow-up. Compared to baseline assessments and the control condition, athletes who participated in *Help Out a Mate* reported increased intentions to provide support to peers alongside positive attitudes about their capacity and willingness to recognize mental health problems in oneself and others.

Preliminary evidence from *Help Out a Mate* also highlighted the potential to consider team environments and related group processes when developing mental health literacy interventions. Beyond evidence that interventions involving peers have resulted in positive mental health literacy outcomes (e.g., [7, 11]), adolescents described their team as a valued place to engage in mental health discussions because of perceived trust and similarity with teammates [16]. Given that team environments can translate to positive outcomes and intervention adherence, it is crucial to leverage team environments to promote mental health.

### Harnessing small group processes

Beyond being a peer setting through which athletes prefer to receive interventions, sport teams entail numerous small group processes that can be harnessed. Researchers have indeed reported that when youth perceive their team as a positive environment (e.g., high group cohesion), they tend to report outcomes such as confidence and social connectedness [17]. Team processes can also be leveraged in interventions to achieve aims that fall beyond merely strengthening bonds within teams. For example, Kroshus and colleagues [18] described how interventions seeking to increase willingness to report concussion symptoms could leverage group norms, by having coaches, parents, and teammates endorse specific behaviors. Related to mental health, group processes associated with norms and identity could be harnessed in team-based interventions.

*Social norm*s refer to informal rules that guide individual behavior in groups [19]. Social norms are commonly the target of interventions related to health behaviors because they are so readily shifted within small groups. For instance, McAlaney et al. [20] discussed the development of a social norms approach to drug education, aiming to reduce misconceptions around others’ consumption. Along with social norms, being a member of a small group tends to entail social identities that more abstractly provide the sense of what it means to ‘be’ a group member. *Social identity* is the portion of an individual’s self-concept which derives from knowledge of group membership [21]. Adolescent athletes rapidly develop social identities within sport teams that are associated with psychosocial benefits [22]. For instance, a recent correlational study revealed adolescents experienced enhanced subjective wellbeing and reduced psychological distress when they perceived high autonomy in their sport involvement–but only for athletes with strong sport team identities [23].

Norms are a potential target for mental health interventions because small groups likely construct behavioral norms regarding supportive behaviors among teammates as well as personal disclosure. Social identities also work alongside social norms to amplify their salience, whereby strong identities strengthen the pressure for members to align their behaviors with those of the group [24]. It therefore seems likely that an ideal mental health literacy intervention delivered in sport teams may integrate strategies to reshape what adolescent athletes believe that being member of their group entails (norms) while reinforcing the athletes’ identity as a team member (identities).

### The current study

It is critical to develop interventions that expand adolescents’ capacity to seek help or assist peers experiencing mental health problems. Team environments are both desirable and efficient settings for delivering interventions with youth, while also including group processes that can be harnessed to amplify intervention messages. The purpose for the current study was to report on the design and delivery of a novel team-based mental health literacy intervention and to conduct a primary evaluation focused on feasibility and acceptability. This study specifically reports on a pilot study of the *Team Talk* intervention–a brief workshop-based intervention to increase adolescent athletes’ mental health literacy while linking such messages to group norms and identities.

#### Feasibility and acceptability

Our approach to evaluating feasibility and acceptability for pilot sessions involved gathering information throughout recruitment and implementation, along with feedback from athletes, coaches, and parents. Feasibility and acceptability trials often focus on one or more topics outlined by Bowen and colleagues [25], such as (a) acceptability: how potential or actual participants react to an intervention, (b) demand: interest-in and use-of intervention activities, (c) implementation: extent to which activities are used as planned, (d) practicality: intervention use when resources are limited, (e) integration: whether system-level change to policy or practice is needed for intervention to be used, and (f) limited efficacy testing: testing proposed effects with constraints to sample and design that limit generalizability. Our approach resembled that of recent formative or process evaluations reported in relation to mental health in sport (e.g., [26]) as well as physical activity (e.g., [27]). We sought to identify what was most effective, for whom, and through which contextual conditions through an evaluation involving the experiences of those involved in interventions as well as by documenting the recruitment and implementation of the intervention.

A feasibility and acceptability focus for the trial was necessary because: (a) the peer-based strategies used in this intervention were unique compared to past research, and (b) we delivered *Team Talk* within a novel context. Regarding context, although numerous youth development interventions have been studied within the United States, existing evaluations of sport-based mental health interventions were conducted in settings outside of North America. This distinction is relevant, considering that competitive sport systems within the United States often differ from those of countries like Australia or the United Kingdom (e.g., [28, 29]).

Whereas our pilot trial design lacked a comparison condition as well as longitudinal follow-up, the evaluation of certain proximal outcomes that are potential explanations can still deliver insights (i.e., limited efficacy testing [25]). Notably, process or formative evaluations can help understand why specific intervention components are effective or ineffective by examining proximal mechanisms. A supplemental purpose therefore focused on whether the intervention demonstrated usefulness relating to variables that have potential to shift within a brief timescale as evidence that the intervention activities are touching-upon anticipated effects. First, efficacy in responding to mental health problems was targeted as an intrapersonal process would increase throughout the workshop session. Second, team social identification was assessed to examine the extent the intervention strengthened athletes’ connection to their group.

## Method

We used a mixed-methods design and collected data through recruitment and implementation logs, surveys, and interviews. Whereas acceptability was measured directly from athlete participant surveys and through interviews, the RE-AIM framework [30] informed our approach to identify relevant indices that may relate to feasibility. Researcher logs and participant surveys targeted reach (e.g., proportion of eligible teams and athletes involved in the trial) and implementation (e.g., whether facilitators delivered content as intended). Interviews targeted similar components, along with adoption and maintenance in terms of the potential for clubs and coaches to deliver this intervention on their own. Participant surveys were also designed to examine effectiveness in influencing presumed proximal mechanisms, including efficacy in responding to mental health problems and social identification. Mental health literacy measures were not delivered before and after the intervention because we anticipated that more specific shifts related to literacy would emerge over a longer time span than the brief before/after assessment periods in the current study.

### Participants and sampling

Participants were initially recruited at the level of clubs and teams, whereby male and female adolescent sport clubs were contacted using publicly available e-mail addresses and phone numbers. We sought competitive teams that trained at least twice per week and competed regionally, with the presumption that the workshop would be most potent when team members interact frequently. Although teams were recruited from several sports, we focused on private competitive lacrosse teams. Prior to commencing this research, we acquired approval from the Institutional Review Board for the lead investigator. This research complies with ethical policies declared by PLOSOne. Of note, written consent was gained from parents’ online forms or using pen and paper forms prior to presenting the study to athlete participants. Written assent was completed by all intervention participants using pen and paper forms.

Eleven adolescent sport teams from the Northeastern region of the United States participated, including six lacrosse teams, four field hockey teams, and one wrestling team. A total of 174 adolescent athletes (84% female) belonged to these 11 teams and participated in the intervention. The sample of interest in the current study when relating to survey measures focused on a subset of 119 participants from across the 11 teams who completed the intervention, but who also chose to complete the survey following the intervention. Participants ranged from 12 to 18 years of age, although most (i.e., 86.6%) participants were from 13 to 16 years of age (*M* = 14.89, *SD* = 1.36). Regarding ethnicity, the majority of participants (i.e., 90%) identified as Caucasian, 3% identified as African-American, 3% identified as Asian, 2% identified as Hispanic, and 2% identified as other. Participants averaged 2.66 years (*SD* = 1.98) with their current club. Follow-up interviews were completed with a subsample of five athlete intervention participants, along with nine parents of athlete participants and two coaches. Female athletes from lacrosse and field hockey teams participated in these interviews. Interviews were conducted by the facilitators who led the intervention (i.e., MBE and MP).

### Procedure

#### Intervention design: *Team Talk*

**Fig 1:**
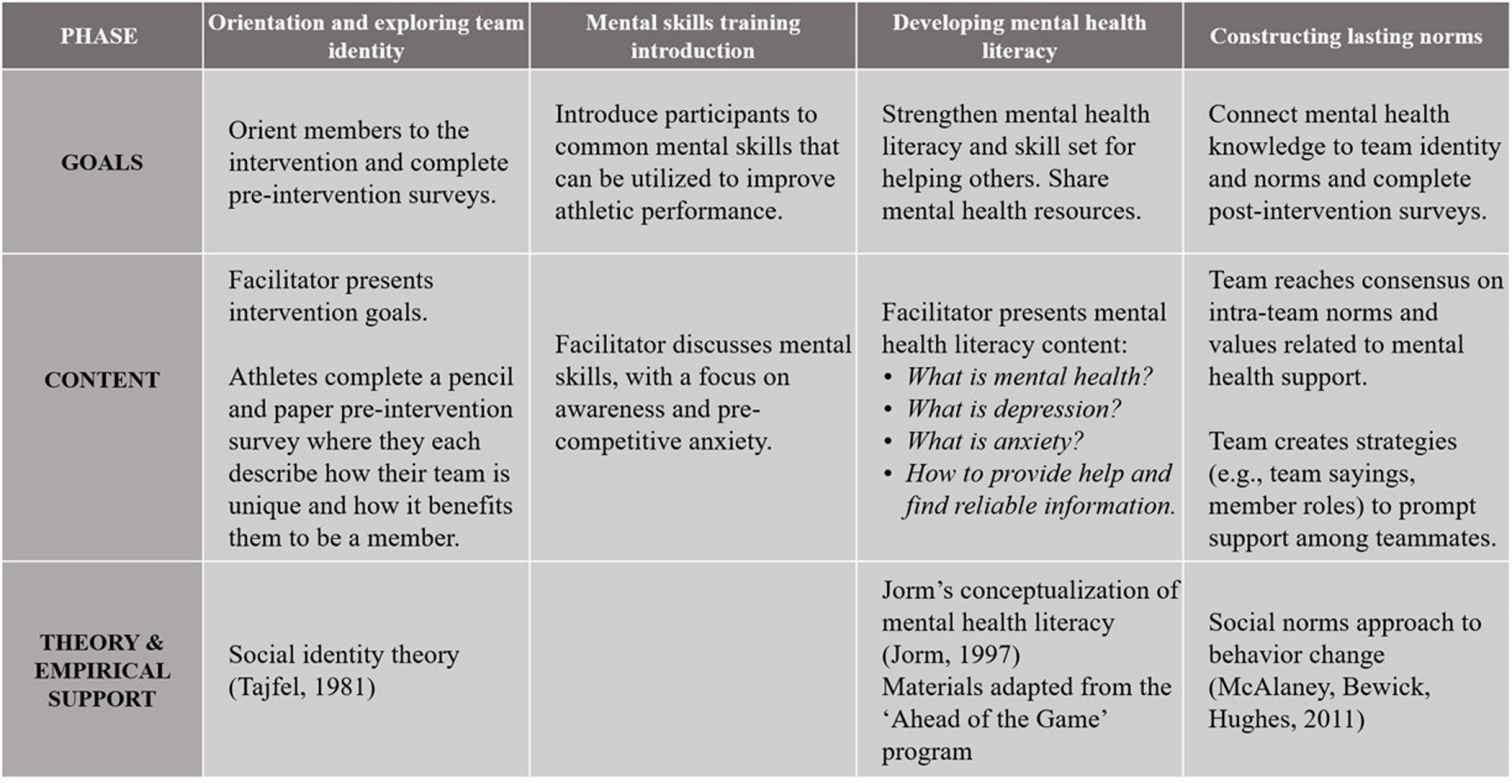
Outline of intervention goals, content, and support.

The *Team Talk* intervention was inspired by the *Help Out a Mate* workshop within the multi-component intervention delivered by Vella and colleagues to adolescent male athletes in Australia [11]. Aligning with the concept of mental health literacy and structured from Mental Health First Aid curricula [13], the broad goal of *Help Out a Mate* is to increase participants’ ability to recognize mental health problems and assist peers experiencing such problems. The *Team Talk* intervention was nevertheless delivered in a novel context (i.e., United States; inclusive of female teams), included a focus on group processes, and integrated resources that were directly relevant to the regional context (e.g., informative websites and helplines). Relating to group-based components, athletes discussed what made their team unique as well as norms or behaviors related to peer support. Mental skills training content (e.g., coping with pre-competition anxiety) was also integrated in *Team Talk*, as a general introduction to sport psychology as a sport science. Fig 1 offers a schematic of all content covered during the workshop, focused on four main components in the following order: (a) orientation and social identity, (b) mental skills training, (c) mental health literacy, and (d) identity and team norms discussion.

#### Intervention delivery

The intervention was delivered by two facilitators: A faculty member (MBE) and a graduate student (MP). Both facilitators attended mental health first aid certification training prior to delivering workshops. A research assistant contacted sport clubs through email, social media, and phone calls. Leaders of clubs that agreed to participate were contacted to arrange a time and location for workshops targeted toward individual teams of athletes. Workshop sessions took place at sites that were convenient for each respective club, including available classrooms, conference rooms, and open space at training facilities.

Prior to initiating the workshop session, athletes filled-out a pre-survey that included: (a) open-ended items prompting athletes to reflect on their team identity, along with (b) survey items regarding social identity strength and self-efficacy (i.e., baseline values). Following these initial questions, one facilitator presented the material while the other facilitator reviewed team identity item responses, with the goal of crystallizing themes from the team’s responses and summarizing these to guide team discussion later in the workshop. The workshop session was guided through content presented via PowerPoint slides or printed 36” X 42” posters when electronic presentations were not possible. Athletes were also provided with a worksheet related to mental skills training as well as a resource card with names and contact information for regional and national resources, such as help lines, mental health service providers, and sport-specific organizations (e.g., ‘SafeSport’; safesport.org).

In addition to content presented by facilitators, discussion was encouraged among team members to elaborate on key concepts. This was especially important for the final component where teammates discussed pre-survey responses regarding team identity. After introducing the most common responses and reading de-identified responses, the workshop facilitator prompted team members to elaborate on their perspectives. Additionally, participants were prompted to identify team norms regarding the behaviors for members to support one another. Examples of responses to the team discussion activity included: (a) identifying team protocol for checking-in on teammates, (b) identifying a ‘buddy’ pairing for each person on the team or a team leader with that role, and (c) identifying existing supportive team sayings.

Athletes completed surveys after the intervention session that included demographics (e.g., sex, age, ethnicity, years of involvement), social identification, self-efficacy, and acceptability of intervention components. Researchers also invited parents, coaches, and athletes to participate in follow-up one-on-one phone call interviews to discuss the intervention. Athlete interviews included questions on further intervention feedback and focused on the extent to which participants viewed peers in sport as sources of support. Parent and coach interviews entailed describing the goals of the intervention and asking them to consider its adoption and implementation within clubs. Interview participants were compensated with a $20 gift card.

### Measures

#### Acceptability (athlete)

The post-intervention survey included questions to obtain feedback from participants on a Likert-scale, ranging from 1 (*strongly disagree*) to 7 (*strongly agree*). Each of the seven items can be found in Table 1. Although items were developed for the purpose of the current research, items were adapted from the AFFIRM acceptability scale which includes items on intervention appropriateness, enjoyment, and usefulness [31].

**Table 1:** Means (SD) of the seven acceptability items.

Acceptability Item	*M*	*SD*
1. I learned a lot from the *Team Talk* workshop	5.76	1.27
2. I had a chance to participate and discuss my thoughts	5.67	1.61
3. The *Team Talk* workshop was well-organized	6.48	0.99
4. I can use what I learned to help myself or my teammates	6.43	1.04
5. This workshop will help my team be more close-knit	5.84	1.23
6. The *Team Talk* workshop was enjoyable	6.10	1.17
7. I enjoyed the *Team Talk* presentation slides	5.93	1.29

#### Social identity strength (athlete)

The Social Identity Questionnaire for Sport was used to assess social identification strength with one’s team [32]. This scale includes nine items completed on a Likert-type scale ranging from 1 (*strongly disagree*) to 7 (*strongly agree*) and has been supported through validation research conducted with adolescent athletes [32]. Given that we intended to examine how athletes’ perceived identity strength differed pre- and post-survey, separate social identification items were distributed into pre- and post- surveys. A split scale approach (4 items at pre-intervention; 4 items post-intervention) was chosen to reduce participant fatigue or item recall from completing identical items before and after the workshop. One item was removed to provide an even number of items (i.e., *I feel strong ties to other members of this team*), and remaining items were allocated into either the pre- or post-survey. The four items assigned to each timepoint are provided in online supplementary material. Mean scale scores were computed for each time point.

#### Self-efficacy: Recognizing problems and providing help (athlete)

Self-efficacy was measured with respect to recognizing mental disorder symptoms and helping teammates experiencing mental health problems. Two items were included at pre- and post-surveys and were completed on a scale from 0 (*not at all confident*) to 100 (*very confident*). Athletes first read a 50-word long fictional scenario where a teammate was acting in ways that were not normal for them and that they overheard the teammate saying that he or she felt ‘down’. Athletes then rated their confidence that they could recognize the athlete was experiencing mental health problems (item 1), and their confidence in asking teammates how they are doing (item 2). While we developed these items for the purpose of this study, they were adapted from Hurley and colleagues [12].

#### Researcher logs

A further source of data were the researcher logs. Regarding recruitment, clubs that were contacted to participate in the intervention were recorded in an excel spreadsheet by a research assistant, along with details regarding responses of clubs. Regarding implementation, the lead author completed a post-workshop journal following each session that included general experiences (e.g., group engagement, perceived clarity of delivery, any challenges during delivery), and structured prompts (e.g., workshop venue, duration of delivery).

### Analyses

Analyses focused on several key goals of this study: (a) summarizing researcher logs regarding implementation and reach, (b) descriptive analyses involving acceptability items, (c) analyses examining change in social identity and efficacy items, and (d) thematic analyses of qualitative interviews. Regarding descriptive analyses to evaluate participants’ responses regarding acceptability, these analyses focused on reporting average (*M*, *SD*) perceptions across the sample regarding acceptability, and through t-tests to identify mean differences between the items. We also examined correlates of acceptability items to identify potential differences in acceptability using independent samples t-tests and bivariate correlations. Quantitative analyses focused on evaluating preliminary effectiveness. Social identity and self-efficacy responses were contrasted using dependent samples t-tests (i.e., pre-/post-intervention comparison).

Qualitative interviews were thematically analyzed [33–35] using NVivo software. Although this report provides limited opportunity to describe methodology and establish rigor, we adopted a generic and descriptive qualitative approach [34] and used reflexive thematic analysis [35] underpinned by a critical realist ontology underpinned by essentialism. What this means is that we adopted an established approach toward thematically analyzing the data alongside a perspective of taking participants’ meaning and reflections on the intervention literally, without applying a particular philosophical or critical lens to interpret statements or themes. When constructing and reporting themes for this report, the authorship team focused on insights from participants with the greatest potential to improve adoption and maintenance of intervention activities within their organizations as well as the relevance of teammates for athlete mental health. We therefore prioritized themes focused on participant experiences with the intervention, reflections on the significance of the issue at hand, and reflections on how participants viewed the consequences or impacts of the intervention.

The lead author from this report reviewed all transcripts and coded each athlete, parent, or coach transcript while adopting the six-phase approach advocated by Braun and Clarke [33, 35]. The lead author familiarized himself with transcripts and intervention notes (Phase 1) before generating initial codes by open-coding each participant statement according to their stated meaning (Phase 2). This was followed by generating themes focused on our goals entering-in to the analyses (Phase 3), and then reviewing the resulting themes as an authorship team (Phase 4). We then defined and named each theme (Phase 5) prior to producing the final report (Phase 6).

All quantitative data are provided as a raw dataset within an OSF page (https://osf.io/wvbp7/?view_only=1ab7bcf295ac408388d6141e46e220be). Qualitative transcripts or excerpts were not included within the publicly accessible data because participants reported on their experiences in the intervention alongside teammates; even though transcripts were deidentified, participants could be identified by others (e.g., teammates; coaches) who may recognize any shared experiences or narratives.

## Results

### Feasibility: Recruitment and implementation

Despite initial interest from many clubs (i.e., positive initial e-mail responses), there was difficulty in committing clubs to intervention sessions. Among clubs contacted via e-mail or phone, 8% of clubs replied to recruitment with only 1% scheduling an intervention. In addition to tracking club-level adoption of the intervention approach, we also tracked involvement of coaches/teams within those club settings as well as athletes within teams. In total, seven clubs decided to adopt the intervention. Across these seven clubs, we had 73% adoption from specific teams. Specifically, fifteen teams were eligible for intervention involvement and eleven teams participated in the intervention (i.e., four clubs had two participating teams; three clubs had one participating team). Coaches from the four teams that did not participate reported lack of available time to complete the intervention. Regarding athlete-level recruitment, all 174 present team members completed the intervention activities, and 119 (68%) of participants chose to complete surveys after completing the intervention session.

Several workshops were scheduled during a large multi-day tournament, with teams completing the workshop during their down time (i.e., before or after their daily games). Although this was convenient for parents and athletes, two workshop sessions were constrained regarding the time available because of scheduling challenges with delivering sessions amidst team activities. Other workshops were delivered before or after weekly team practices. Interventions ranged from 40 to 75 minutes–with an average around 56 minutes (*SD* = 11 mins). Coaches observed nine of the eleven sessions. Although comparable interventions excluded coaches (see [7]) the inclusion of coaches was integrated into the intervention plan early-on because many coaches of private clubs would only accept the intervention if they could also observe. Although this may present barriers, coach inclusion could be a strategy to ensure that intervention messages are sustained within groups.

### Acceptability: Athlete survey responses

The mean score and standard deviation for each acceptability item is illustrated in Table 1. Participants reported relatively positive perspectives: Although no clear reference value or comparison is available regarding acceptability, all values were near the high end of the rating scale for items. Participants especially reported having learned a great deal, feeling like they had the potential to apply their learning, and general enjoyment during the session.

Recognizing that athletes nevertheless varied regarding these perceptions, we also examined bivariate correlations (see online supplementary material) regarding demographic participant characteristics (e.g., years on team) along with aspects related to intervention implementation (i.e., ordering of intervention session within the current trial). Whereas no significant correlations with demographic variables were identified, acceptability items were significantly correlated with both session ordering and duration. Regarding the order of the interventions, those who participated in interventions closer to the end of the trial were more likely to perceive that they learned a lot (*r* = 0.21, *p* = .02), had a chance to participate (*r* = 0.19, *p* = .04), and felt that they could use what they learned (*r* = 0.24, *p* = .01). Although intervention content remained consistent throughout the study, the shift in acceptability relating to the intervention session order may signal learning on the part of facilitators. Regarding duration, those who participated in longer sessions also perceived that they had a chance to participate (*r* = 0.24, *p* = .09), the intervention was organized (*r* = 0.23, *p* = .01), and the intervention would help their team be more close-knit (*r* = 0.28, *p* = .02). Participants in longer sessions may have experienced more opportunities to participate and communicate with teammates.

### Preliminary effectiveness: Athlete survey responses

Recall that social identity strength and efficacy were reported before, and immediately following, sessions to help identify plausible shifts in intervention targets. Paired t-tests revealed a significant difference between social identity scores, *t* (114) = -4.48, p < 0.001 (*d* = 0.35). When compared to baseline responses regarding social identity strength (*M* = 6.08, *SD* = 0.79), participants reported stronger social identities at follow-up (*M* = 6.34, *SD* = 0.71). Regarding efficacy perceptions, significant differences were not identified in relation to symptom recognition or helping teammates. Related to recognizing symptoms of mental disorders, participants reported an average score of 71.78 (*SD* = 19.09) in the pre-intervention survey and a score of 77.56 (*SD* = 17.39) in the post-intervention survey; these values did not significantly differ(*d* = 0.32, *p* = 0.06). Regarding efficacy for providing help, participants reported an average of 85.25 (*SD* = 17.66) and 85.08 (*SD* = 17.74) on the pre- and post- intervention surveys, respectively, and these values did not differ (*d* = 0.01, *p* = 0.91).

### Qualitative interviews

Athlete, parent, and coach participants generally described positive attitudes toward intervention acceptability during phone interviews. Particularly for parents and coaches, responses focused on the necessity of mental health literacy interventions within the daily contexts of their sport clubs and homes–whereas athlete interviews focused more on their experiences during the workshop itself. Our interviews focused on addressing the acceptability of the intervention implementation as a whole, and less on specific intervention components, because: (a) recruitment challenges meant that acceptability was a foremost hurdle, and (b) parents and coaches were best suited for these more general reflections. Resulting themes focus on perceptions of acceptability in the participants’ sport contexts, potential adaptations, and the role of teammates as resources.

#### Acceptability

Athlete participants discussed enjoying the intervention and how they developed knowledge that could be directly applied to support teammates. For example, one athlete discussed learning the importance of checking in with teammates:

“I think it [the intervention] makes us realize that even if someone’s acting perfectly fine in practice, we have to still check in on them to make sure they are doing okay and not just acting okay or something.”

Particularly because this response was stated by the athlete in the days following the intervention, the similarity of the response to the intervention’s goals demonstrates that this participant absorbed the general goal of their workshop. Additionally, an athlete mentioned the importance of discussing mental health, given the topic’s lack of attention:

“I thought it was good to talk about those topics and kind of take a break from the normal practice and talk about things that are going on that I feel like aren’t really talked about much.”

This point highlights the demand for mental health literacy interventions alongside adolescent athletes’ willingness to discuss mental health problems.

Parents and coaches focused on the demand for mental health literacy interventions, discussing the prevalence of adolescent mental health problems. For example, one coach anecdotally described a sense that widespread anxiety among adolescent athletes seemed to increase throughout 18 years of coaching. Through these observations, this coach realized the urgency for strategies that can mitigate mental health problems. Additionally, one parent described that many adolescents do not recognize the prevalence of mental health problems and indicated that interventions during the instability of adolescence are critical:

“Kids need to know that they are not alone. So, I think doing it during adolescence when, you know, puberty and all the other physical factors are being taken into account, I think that’s really important and I think we should do that more with kids in that age.”

#### Enhancing adoption

Despite the overall positive feedback about the demand and acceptability of the intervention, participants reflected on potential adaptations to intervention delivery. Aligning with the challenges from the research team in recruiting participants, coaches and parents both reflected on competing demands on athletes’ time and strategies to maintain mental health literacy. The lack of available time highlights the need for facilitator flexibility. Additionally, athletes discussed how potential intervention benefits could be maximized if presented early in the sporting season, to help shape the team environment.

Parents discussed how they mainly had opportunities to discuss their child’s workshop experience with them after the session took place–and that efforts to prepare adolescents in advance could provide an opportunity for more rich participation during session. For example, one parent suggested providing some of the participation questions a week or so prior to the intervention for athletes to prepare. Athlete participants from two workshops also commented on how the workshop was useful, but they were concerned that the key messages may not be maintained within the groups. One athlete described how check-in meetings may help maintain the key message and provide opportunities for athletes and coaches to provide support:

“I feel like maybe if there were multiple meetings and maybe like once a month or once every two months, just like a quick hour to check-in and see how everyone’s doing.”

Coaches and parents offered additional recommendations, such as providing resources for coaches and parents that could ensure that messaging is consistent across the organization.

#### Peers as resources

Participants commented on ways that teammate interactions were perceived to have changed after the intervention, in addition to more generally reflecting on the role of teammates in recognizing to mental health problems. Athletes and coaches thus reflected on how the intervention provided an opportunity to strengthen existing bonds with teammates and improve team members’ willingness to share their feelings with teammates. One coach described how the intervention prompted teammate communication:

“I think 95% of that group walked out of there like, ‘okay this was good for us, that was really awesome’ and I think they even gained more knowledge on how to best communicate with their teammates.”

Athletes also described how teams can be an effective target for a mental health intervention. Interestingly, athletes rarely described the aspects of teams that were the focus of the current intervention, with identity and team norms rarely being discussed relative to mental health. In contrast, athletes focused on social support and emotional regulation of others. For example, one athlete discussed how her teammates could be a resource:

“They always try and make my day better. Especially if I’ve had a rough day at school and stuff like that, they’ll always be there to cheer me up and make me laugh.”

This quote reflects on how adolescents on her team sought opportunities to check-in on her and provide support–even before participating within the workshop.

Athlete participants also described seeking support from teammates because they felt similar and could better-relate. One athlete participant acknowledged that they preferred their discussions with teammates, and this sentiment was shared by a parent: “So, it’s … a great thing that she has this outlet [beyond her parents], and that in the same time you’re teaching them ways to assist themselves and ways to assist their peers.” Thus, teammates were recognized as resources for mental health promotion.

## Discussion

This study evaluated the feasibility and acceptability of an adapted mental health literacy intervention specifically for adolescent sport teams. The *Team Talk* intervention focused on increasing athletes’ mental health literacy and generating supportive small group environments via identities and norms. The current study revealed challenges with recruitment and identified the potential to enhance the quality of how mental health literacy content is promoted among adolescents. Club-level adoption of the intervention was low, with difficulty recruiting clubs for intervention delivery. This signals that feasibility of the intervention–as it is currently designed–is low when considering similar contexts including competitive adolescent sport clubs and delivered as team-level workshops. Despite low club-level recruitment, the coach- and athlete-level recruitment was descriptively higher. Interviews and surveys from athletes, parents, and coaches through interviews and surveys also provided preliminary evidence for acceptability despite our low club-level recruitment. Our discussion reflects on these challenges to feasibility, followed by considering results from surveys and interviews with athletes, parents, and coaches.

### Recruitment and feasibility

Challenges to club-level recruitment and implementation from this investigation could indicate low levels relating to one or more aspects of feasibility. Recall, specifically, that this intervention was delivered within privately-run and competitive program contexts at a developmental level. When considering low club-level recruitment, we looked toward the operationalization of feasibility from Bowen and colleagues [25] that describes facets like demand, practicality and integration.

We do not anticipate that demand is a primary concern for feasibility. Existing literature indicates there is high demand for mental health interventions in sport programs around the globe (e.g., [12, 16]), and our interviews with athletes, parents, and coaches supported this expectation. The coach- and athlete-level recruitment rates were also high, further signaling that a demand exists. Meanwhile, we expect that practicality and integration are key facets to consider. Practicality relates to whether the intervention can be delivered when resources are constrained, and emerged as a challenge informally when contacting coaches and when conducting interviews. The club settings that athletes belonged to were restricted to specific seasons (e.g., summer) and included pre-elite athletes often located at a distance from their club, so athletes had limited weekly contact hours within their club setting. As a result, dedicating a team session involvement in this intervention presumably took-away from other team activities. Integration refers to whether system-level change to infrastructure or policy is needed to incorporate intervention. We delivered the intervention within a fractured sport context where governing bodies had limited influence on how programming is delivered, when compared to other countries or settings where comparable interventions have seen success [28, 29]. Even though we did not track club reasons for low intervention involvement, and so any attributions to specific facets of feasibility are speculative, we explain our low recruitment to practicality and integration.

One message from the low recruitment could be that team-based interventions are not an ideal fit for the sport context. However, it could also be useful to consider how our approach contrasts with successful examples of comparable interventions. For example, we directly recruited clubs and coaches rather than using a top-down approach through a broader governing body. This meant that individual contacts with coaches and clubs were necessary–making recruitment more demanding–and that we were unable to use ‘champions’ within the community. Vella and colleagues [26] experienced similar recruitment challenges during an initial phase of their intervention and adapted their approach to integrate top-down recruitment (i.e., champions who are club leaders) alongside bottom-up strategies (e.g., working with clubs or teams to modify the intervention activities to suit), and employed a number of club engagement officers focused on forging club connections. Similar adaptations to intervention implementation may aid in feasibility, including: (a) embedding mental health literacy content within other activities in teams (e.g., mental skills training; injury prevention), and (b) partnering with governing bodies, as reported in the *Ahead of the Game* intervention to extend reach [11].

One additional observation is that it is plausible that the recruitment challenges in our studies were not unique to our context but, instead, were brought to light because feasibility was a specific goal in this research. Feasibility studies have traditionally been uncommon in sport studies and past youth sport research has rarely documented the process of implementation. As one example relevant to recruitment challenges, Evans and colleagues reviewed interpersonal coaching interventions in youth sport and observed that: (a) fewer than 20% of studies reported recruitment rates, and (b) none of the studies reported their recruitment relative to comparable target populations [36]. Therefore, there are relatively few benchmarks to understand typical uptake of interventions and to recommend strategies to increase reach. From a hopeful standpoint, programs of sport research are emerging that target knowledge translation, the demands and interests of sports stakeholders, and implementation. One example of emerging research in this domain involves recent interviews with coaches and sport organization leaders to examine the types of knowledge they use in daily practice and to consider changes that need to be made in communicating academic findings [37].

### Acceptability, effectiveness, and intervention experiences

Responses from athletes, parents, and coaches through interviews and surveys provided preliminary evidence for acceptability despite our low club-level recruitment. Feedback from participants included positive evaluations of intervention components and descriptions of the necessity for mental health interventions during adolescence. Participants also identified more strongly with their team following the intervention compared to baseline, supporting the potential value of team identity as a target. We focus our discussion on how this research informs the future design of sport-based mental health literacy interventions, along with theory about peer relationships.

This mixed methods study revealed promising results for researchers interested in leveraging sport as a catalyst for mental health promotion. At a proximal level, this study revealed quantitative effectiveness via descriptively high acceptability ratings. Athletes, coaches, and parents also more generally perceived a demand for mental health literacy interventions. We nevertheless note that a key limitation was that we did not incorporate a multi-dimensional measure of mental health literacy. Whereas self-efficacy for identifying and responding-to mental health problems represents a subcomponent of mental health literacy, tools such as the mental health literacy scale [38] are critical for examining responses across the dimensions of this construct (e.g., ability to recognize disorders; knowledge of resources; knowledge of self-treatments). This measure would be critical to include in future longitudinal tests of interventions like Team Talk.

Focusing on proposed group-based strategies to amplify interventions in teams, we identified that athletes reported stronger social identification with their team following the intervention. Given the link between sport team identity and adolescent mental health [23], promoting social identity among teams may be an effective strategy to enhance mental health on its own. Therefore, youth sport leaders can regularly integrate identity-fortifying strategies into their daily team activities. We anticipate that the prompts used in this study for participants to write-about and discuss what made their team unique and important could be useful in other interventions to influence identity and group cohesion–alongside established team building approaches to draw members together and reinforce the salience of the group [39–41].

Findings from this study also have implications that extend generally to strategies to influence group environments. Notably, athletes often described enjoying the written and discussion-based activities that asked athletes to reflect on their team identity and why they benefit from involvement–and this approach resembles personal disclosure team-building activities (e.g., [41, 42]). The *personal-disclosure mutual-sharing* approach is described as a team-building activity where a facilitator leads team discussions about personal features such as values and meaning behind sport involvement. We nevertheless note caution regarding disclosure. Dunn and Holt [42] noted that disclosure activities may make athletes feel pressured to disclose personal experiences within a group, and that other team members may make inappropriate use of disclosed information. Similarly, researchers studying types of adolescent preventive interventions emphasize that facilitators need to be prepared to manage risks of disclosure [43]. A critical component of interventions such as *Team Talk* thus involves setting expectations before conducting sessions, finding ways to ensure voluntary workshop participation, and sensitivity toward adverse responses to mental health discussions.

Compared to the significant change in identity perceptions, we did not identify significant differences in athletes’ efficacy to recognize mental health problems in teammates and their intentions to provide help to teammates. This finding is contrary to findings of past research, including a controlled trial with the *Help Out a Mate* intervention which reported enhancements in adolescent athletes’ knowledge of mental health problems and fostered positive attitudes following participation in the team-based component [7]. Non-significant effects in our study may be related to how participants reported relatively high perceptions of efficacy at baseline, that our study was underpowered to detect a moderate effect, and that the efficacy measure was brief. Nevertheless, it is critical to elicit change in these outcomes so perhaps future interventions will include additional content to specifically target self-efficacy.

It is also plausible that our intervention did not include an adequate dose of mental health content to shift efficacy perceptions. Variability in dose and exposure to intervention components are important to consider when examining both reach of an intervention as well as effects relating to outcomes like efficacy or knowledge. Regarding intervention dose, the current intervention was a single-session intervention that is relatively brief and time-limited. It took place once during the season and, while workshops were approximately one hour in duration, the core mental health literacy content was often constrained to thirty minutes when paired with team-based and sport performance content. Whereas extending contact time could be one adaptation (e.g., multiple sessions), participants also provided feedback that they would prefer additional access to materials to support the intervention activities. Multiple modes of intervention content may therefore be critical to further development of similar interventions. Research is required to determine the effectiveness of online resources for participants to explore material further, as is research determining the most effective way to distribute resources. One particular approach may involve resources to enable coaches to deliver mental health literacy activities within their teams, such as online modules to increase coaches’ mental health literacy and instruct how to facilitate a discussions within their own team to facilitate social support. Research has also demonstrated the utility of coach facilitators. For example, a program to prevent substance use involved coach-led sessions conducted on several occasions during a sport season and was effective at improving athlete attitudes and intentions toward substances [44].

## Conclusion

This mixed-methods study tested the feasibility and acceptability of a mental health literacy intervention designed for adolescent sport teams. We recruited teams for the research by contacting club representatives and resulting recruitment rates were low–signaling that feasibility of the intervention may be limited. Meanwhile, participants rated the acceptability relatively high, and results demonstrate the potential of leveraging group processes embedded in youth sport to promote mental health. Athlete participants, coaches, and parents discussed potential adaptations to enhance the intervention and reflected on the salience of peer relationships in the sport context. Whereas findings from this research indicate that adaptations to implementation are necessary to increase adoption by sport organizations, this research can inform the design of future mental health literacy interventions alongside efforts to foster positive teammate relationships.

## Supporting information

S1 File Contains both supporting tables. (DOCX)
