# Supplementary material for: Developing and evaluating a peer-based mental health literacy intervention with adolescent athletes
Source: PLoS One. 2022 Dec 15;17(12):e0274761. doi: 10.1371/journal.pone.0274761 (PMC9754252; doi:10.1371/journal.pone.0274761)
Supplement: S1 File — (DOCX) [file pone.0274761.s001.docx]

**Electronic Supplementary Material**

**Article:** Developing and Evaluating a Peer-Based Mental Health Literacy Intervention with Adolescent Athletes

**Journal:** PLOS ONE

**Authors:** Michael Panza, Grace Redman, Matthew Vierimaa, Melissa Bopp, Stewart A. Vella, M. Blair Evans

| *Supporting Table 1. SIQS items in pre- and post-intervention surveys.* | |  |
| --- | --- | --- |
| Pre-Intervention SIQS Items | Post-Intervention SIQS Items | |
| 1. I feel a sense of being "connected" with other members of this team. | 1. I find it easy to form a bond with other members of this team. | |
| 2. I feel good about being a member of this team. | 2. In general, I'm glad to be a member of this team. | |
| 3. In general, being a member of this team is an important part of my self-image. | 3. Generally, I feel good when I think about myself as a member of this team. | |
| 4. The fact that I am a member of this team often enters my mind. | 4. Overall, being a member of this team has a lot to do with how I feel about myself. | |

*Note: The following item was removed from the SIQS for the purpose of this study: “I feel strong ties to other members of this team”.*

| *Supporting Table 2. Bivariate Correlations.* | | | | | | | | | | | |
| --- | --- | --- | --- | --- | --- | --- | --- | --- | --- | --- | --- |
| **Variable** | **1** | **2** | **3** | **4** | **5** | **6** | **7** | **8** | **9** | **10** | **11** |
| 1. Order of intervention | - |  |  |  |  |  |  |  |  |  |  |
| 2. Duration of intervention | -0.20** | - |  |  |  |  |  |  |  |  |  |
| 3. Age of participants | -0.66** | 0.24** | - |  |  |  |  |  |  |  |  |
| 4. Years on team | -0.24** | 0.00 | 0.20* | - |  |  |  |  |  |  |  |
| 5. Hours spent with team a week | -0.32** | 0.12 | 0.11 | 0.09 | - |  |  |  |  |  |  |
| 6. A1 (learned a lot) | 0.21* | 0.07 | -0.06 | -0.09 | -0.03 | - |  |  |  |  |  |
| 7. A2 (opportunities to participate) | 0.19* | 0.24** | -0.10 | 0.08 | -0.17 | 0.40** | - |  |  |  |  |
| 8. A3 (well-organized) | 0.23** | 0.23* | -0.15 | 0.00 | 0.08 | 0.44** | 0.43** | - |  |  |  |
| 9. A4 (learned how to help others) | 0.24** | 0.14 | -0.11 | -0.01 | 0.03 | 0.61** | 0.43** | 0.51** | - |  |  |
| 10. A5 (helped us be close-knit) | 0.16 | 0.28** | -0.03 | -0.12 | -0.06 | 0.61** | 0.47** | 0.46** | 0.60** | - |  |
| 11. A6 (enjoyable) | 0.10 | 0.17 | 0.07 | -0.03 | 0.02 | 0.54** | 0.43** | 0.49** | 0.48** | 0.66** | - |
| 12. A7 (enjoyed slides) | 0.16 | 0.07 | 0.05 | -0.06 | 0.06 | 0.54** | 0.31** | 0.48** | 0.49** | 0.54** | 0.74** |
| * p < .05; ** p < .01  *Note: Pearson correlations are reported in this table, with the exception of correlations related to intervention ordering. Because the order of sessions (1-11) was a rank order variable, we report Spearman rank-order correlation statistics (column 1). Also, variables 6-11 refer to acceptability-related items.* | | | | | | | | | | | |
